# Investigating the Effects of Sex Hormones on Macrophage Polarization

**DOI:** 10.3390/ijms25020951

**Published:** 2024-01-12

**Authors:** Sophie Enright, Geoff H. Werstuck

**Affiliations:** 1 Thrombosis and Atherosclerosis Research Institute, 237 Barton Street E, Hamilton, ON L8L 2X2, Canada; enrighsm@mcmaster.ca; 2 Department of Medicine, McMaster University, 1280 Main St. W, Hamilton, ON L8S 4L8, Canada

**Keywords:** macrophage polarization, 17β-estradiol, testosterone

## Abstract

Sex differences in the development and progression of cardiovascular disease are well established, but the effects of sex hormones on macrophage polarization and pro-atherogenic functions are not well described. We hypothesize that sex hormones directly modulate macrophage polarization, and thereby regulate the progression of atherosclerosis. Bone marrow-derived monocytes from adult male and female C57BL/6 mice were differentiated into macrophages using macrophage colony-stimulating factor (20 ng/mL) and pre-treated with either 17β-estradiol (100 nM), testosterone (100 nM), or a vehicle control for 24 h. Macrophages were polarized into pro- or anti-inflammatory phenotypes and the effects of sex hormone supplementation on the gene expression of macrophage phenotypic markers were assessed using RT-qPCR. Inflammatory markers, including IL-1β, were quantified using an addressable laser bead immunoassay. A transwell migration assay was used to determine changes in macrophage migration. Sex differences were observed in macrophage polarization, inflammatory responses, and migration. Pre-treatment with 17β-estradiol significantly impaired the gene expression of inflammatory markers and the production of IL-1β in inflammatory macrophages. In anti-inflammatory macrophages, 17β-estradiol significantly upregulated the expression of anti-inflammatory markers and enhanced migration. Pre-treatment with testosterone enhanced anti-inflammatory mRNA expression and impaired the production of IL-1β. Our observations suggest a protective role of 17β-estradiol in atherogenesis that may contribute to the sexual dimorphisms in cardiovascular disease observed in human patients.

## 1. Introduction

Differences in cardiovascular disease (CVD) onset, presentation, and severity exist between men and women [1]. Premenopausal women have a much lower prevalence of obstructive ischemic heart disease (IHD) and plaque rupture than similarly aged men [2]. Several clinical studies have demonstrated that women with IHD are older and present with more CVD risk factors than men [1,2,3]. Furthermore, while men have an incidence and related mortality of IHD that are twofold greater than for women, the disparity is reduced with age [4]. Regardless, CVD risk increases with age in both men and women [5], which coincides with the reduction in, or loss of, reproductive hormone production [6]. It has been suggested that observed differences in CVD presentation, risk, and severity with age and between sexes may be attributable, at least in part, to sex hormones [7], including estrogen, progesterone, and testosterone.

Preclinical studies have explored the implications of sex hormones, especially estrogen and, to a lesser extent, testosterone, on CVD risk. Estrogen improves vascular function and protects against atherosclerosis in mice [8]. The effects of estrogen on the endothelium are relatively well established. In instances of vascular injury, it has been demonstrated that estrogen promotes re-endothelialization [9], inhibits smooth muscle cell proliferation [10], and attenuates plaque progression [11].

Experimental studies have also suggested a protective role of testosterone and androgen receptors against cardiovascular disease. Testosterone deficiency through castration increased atherogenesis, an effect which was abolished with testosterone supplementation [12]. Furthermore, in male mice, androgen receptor deficiency results in an increased atherosclerotic burden [12]. While testosterone has been demonstrated to act through androgen receptors, it may also exert its athero-protective effects through its conversion into estrogen by aromatase. In male mice, aromatase inhibition blocks testosterone’s anti-atherogenic effects [13]. This effect may be mediated through the modulation of vascular tone because aromatase knockout mice also present with irregular vascular relaxation [14].

Owing to increasing evidence for sex hormone involvement in atherosclerosis, and accumulating observations that connect macrophage phenotype distribution to atherosclerosis severity [15], we propose that sex hormones directly influence macrophage polarization and pro-atherogenic functions. In preclinical in vitro studies of macrophage polarization in murine bone marrow-derived macrophages (BMDMs) and THP-1 macrophages, estrogen has been demonstrated to promote polarization to the anti-inflammatory (M2) phenotype and reduce polarization to the pro-inflammatory (M1) phenotype [16,17,18,19]. Likewise, a smaller body of evidence supports a role for testosterone in the promotion of the anti-inflammatory phenotype [20,21]; however, differences in experimental protocols, including variability in the polarization agents used and the dosages of these agents, the types and dosages of sex hormone treatments, and the number of sample collection times, impede our ability to compare observations from separate studies. Furthermore, very few studies have considered the effects of both estrogen and testosterone on macrophage polarization and functions in the same experimental protocol. Most notably, to our knowledge, no study has connected these observations to the pro-atherogenic functions of macrophages.

We hypothesize that sex hormones directly modulate BMDM polarization and pro-atherogenic function, and thereby regulate the progression of atherosclerosis. This study will directly compare the role(s) of both estrogen and testosterone in BMDM polarization and pro-atherogenic functions in one complete study using one standardized protocol. It will also acknowledge sex as an independent factor to be considered in this context. The goal of this research is to further justify and inform efforts to develop new, and more effective, sex-specific strategies with which to treat patients with CVD.

## 2. Results

### 2.1. Characterization of BMDM Polarization

**Figure 1:**
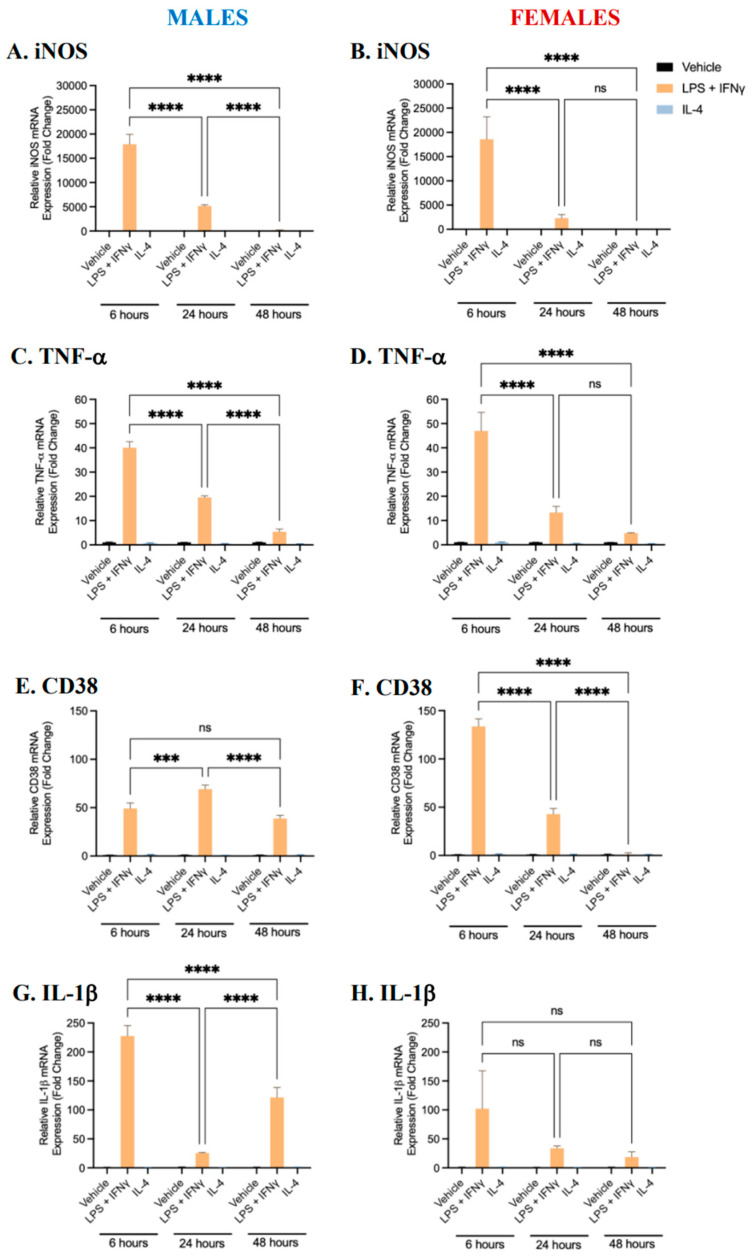
Gene expression of pro-inflammatory markers in male- and female-derived BMDMs. Bone marrow monocytes from male and female C57BL/6 mice were differentiated into BMDMs and treated with LPS and IFNγ, IL-4, or a vehicle control for 6, 24, or 48 h to polarize cells to pro- and anti-inflammatory phenotypes or to maintain an unpolarized phenotype. The gene expression of pro-inflammatory markers was quantified in male (**A**,**C**,**E**,**G**)- and female (**B**,**D**,**F**,**H**)-derived BMDMs using RT-qPCR. Data shown represent the mean ± SEM fold change (2^−ΔΔCt^) in marker expression relative to the reference gene β-actin, normalized to the vehicle control. A two-way ANOVA and Tukey’s multiple comparison test were performed. (n = 4, *** *p* < 0.001, and **** *p* < 0.0001, ns—not significant).

**Figure 2:**
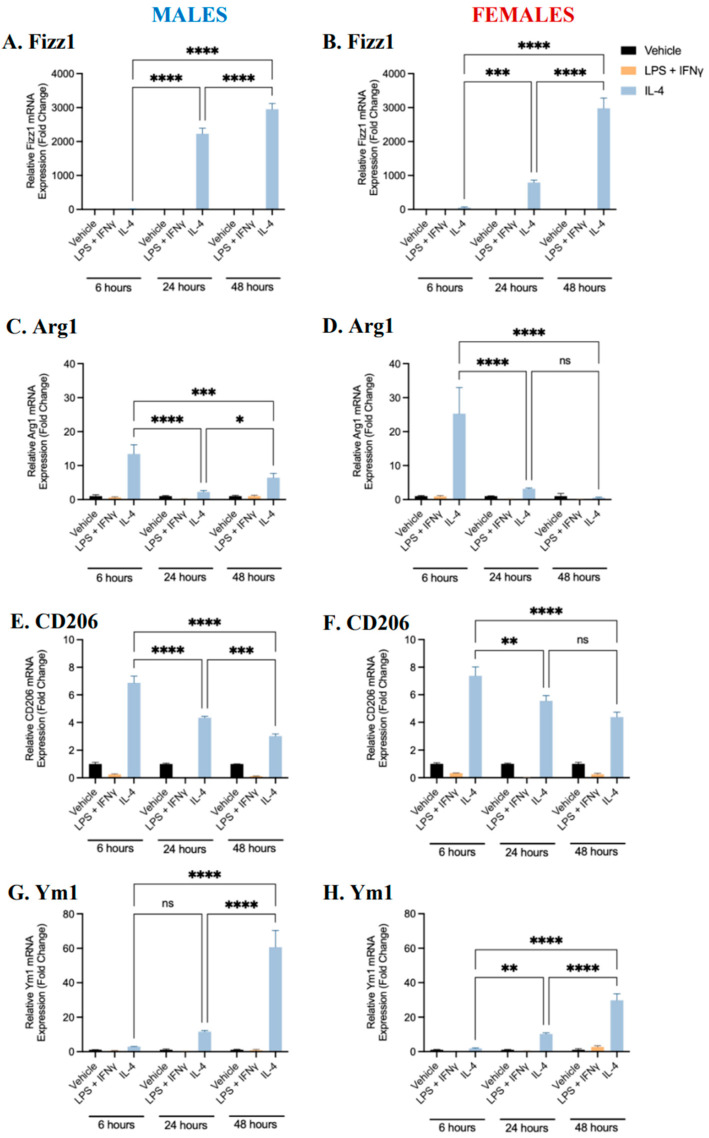
Gene expression of anti-inflammatory markers in male- and female-derived BMDMs. Bone marrow monocytes from male and female C57BL/6 mice were differentiated into BMDMs and treated with LPS and IFNγ, IL-4, or a vehicle control for 6, 24, or 48 h to polarize cells to pro- and anti-inflammatory phenotypes or to maintain an unpolarized phenotype. The gene expression of anti-inflammatory markers was quantified in male (**A**,**C**,**E**,**G**)- and female (**B**,**D**,**F**,**H**)-derived BMDMs using RT-qPCR. Data shown represent the mean ± SEM fold change (2^−ΔΔCt^) in marker expression relative to the reference gene β-actin, normalized to the vehicle control. A two-way ANOVA and Tukey’s multiple comparison test were performed. (n = 4, * *p* < 0.05, ** *p* < 0.01, *** *p* < 0.001, and **** *p* < 0.0001, ns—not significant).

Our laboratory has previously established protocols with which to polarize murine BMDMs into pro-inflammatory or anti-inflammatory phenotypes [22]. To create a baseline phenotypic profile over time for pro-inflammatory and anti-inflammatory BMDMs, male- and female-derived bone marrow monocytes were differentiated into BMDMs in the presence of M-CSF. Differentiated macrophages were polarized into pro-inflammatory (M1) or anti-inflammatory (M2) phenotypes through exposure to LPS (100 ng/mL) and IFNγ (20 ng/mL), or IL-4 (10 ng/mL), respectively, for 6, 24, or 48 h. Controls (unpolarized M0 BMDMs) were treated with the vehicle alone. The gene expression of pro-inflammatory (iNOS, TNF-α, IL-1β, and CD38) and anti-inflammatory (Arg1, Fizz1, Ym1, and CD206) markers was quantified using RT-qPCR [16,17,23,24]. When BMDMs derived from either male or female mice were treated with LPS and IFNγ, the mRNA expression of iNOS, TNF-α, IL-1β, and CD38 was significantly upregulated (Figure 1). The expression of all pro-inflammatory markers, except for CD38 (in males only), was the greatest following 6 h of polarization. Conversely, when BMDMs were treated with IL-4, the mRNA expression of Fizz1, Arg1, Ym1, and CD206 was significantly upregulated (Figure 2). The expression of Fizz1 and Ym1 was the lowest following 6 h of treatment; however, Arg1 and CD206 expression was the greatest following 6 h of treatment. While Arg1 and CD206 expression was also significantly upregulated after 24 and 48 h of polarization, no significant differences in expression levels were observed between these time points in female-derived BMDMs. The data at each time point are consistent with existing data in the literature and signify that treatment with LPS+IFNγ and IL-4 was sufficient to polarize BMDMs to pro- and anti-inflammatory phenotypes, respectively [22,25,26]. Notably, after 24 h, pro-inflammatory markers were significantly upregulated in pro-inflammatory macrophages and anti-inflammatory markers were significantly upregulated in anti-inflammatory macrophages. Thus, polarization for 24 h was selected as an appropriate time frame with which to carry out further analysis.

**Figure 3:**
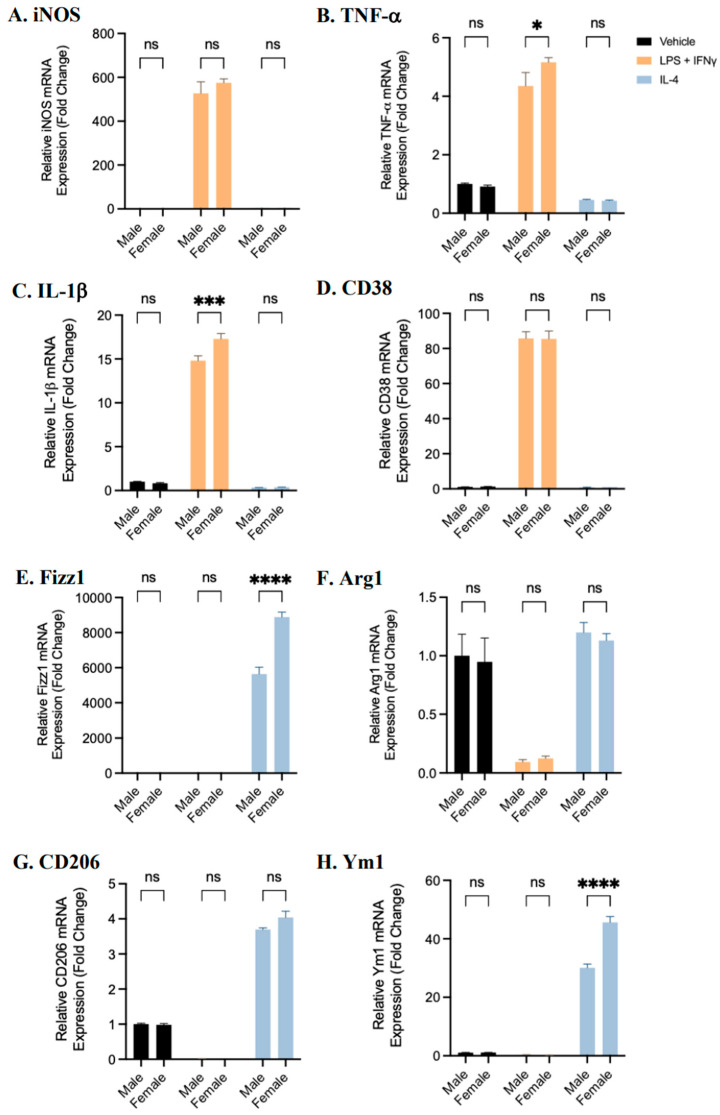
Sex differences in the gene expression of BMDM polarization markers. Bone marrow monocytes from male and female C57BL/6 mice were differentiated into BMDMs and treated with LPS and IFNγ, IL-4, or a vehicle control for 24 h to polarize cells to pro- or anti-inflammatory phenotypes or to maintain an unpolarized phenotype. The gene expression of pro-inflammatory (**A**–**D**) and anti-inflammatory (**E**–**H**) macrophage markers was quantified in female- and male-derived BMDMs using RT-qPCR. The data shown represent the mean ± SEM fold change (2^−ΔΔCt^) in marker expression relative to the reference gene β-actin, normalized to male BMDMs. A two-way ANOVA and Tukey’s multiple comparison test were performed. (n = 4, * *p* < 0.05, *** *p* < 0.001, and **** *p* < 0.0001, ns—not significant).

In the above experiment, differences in BMDM polarization between male- and female-derived BMDMs could not be directly assessed because the sexes were not examined concurrently. To directly investigate potential sex differences the same protocol was carried out, using BMDMs obtained from male and female mice at the same time. Differentiated macrophages derived from male or female mice were polarized to pro- or anti-inflammatory phenotypes, or left unpolarized for 24 h, and the gene expression of pro- or anti-inflammatory markers was quantified. The gene expression of TNF-α and IL-1β in female-derived pro-inflammatory BMDMs was significantly elevated compared to male-derived BMDMs (Figure 3). In addition, female-derived anti-inflammatory BMDMs exhibited significantly upregulated gene expression of Fizz1 and Ym1. These data suggest that female-derived BMDMs may respond more readily to polarization treatments, or that these cells may be more susceptible to environmental stimuli. To investigate whether biological sex affects the gene expression of estrogen or androgen receptors, unpolarized BMDMs were collected and the gene expression of AR, ERα, ERβ, and GPER1 was assessed (Supplementary Figure S1). Female-derived BMDMs had a significantly greater expression of androgen receptors compared to male BMDMs. No sex differences were observed in the expression of estrogen receptors.

### 2.2. BMDM Polarization Is Affected by 17β-Estradiol and Testosterone Supplementation

To determine if BMDM polarization is affected by supplementation with estrogen or testosterone, differentiated BMDMs derived from male or female mice were treated with 17β-estradiol (100 nM) or testosterone (100 nM) for 24 h and then polarized to pro- or anti-inflammatory phenotypes, as described above. The hormone concentrations used are based upon previous publications and preliminary titration experiments [16,27,28]. The mRNA expression of pro- and anti-inflammatory markers was quantified using RT-qPCR.

**Figure 4:**
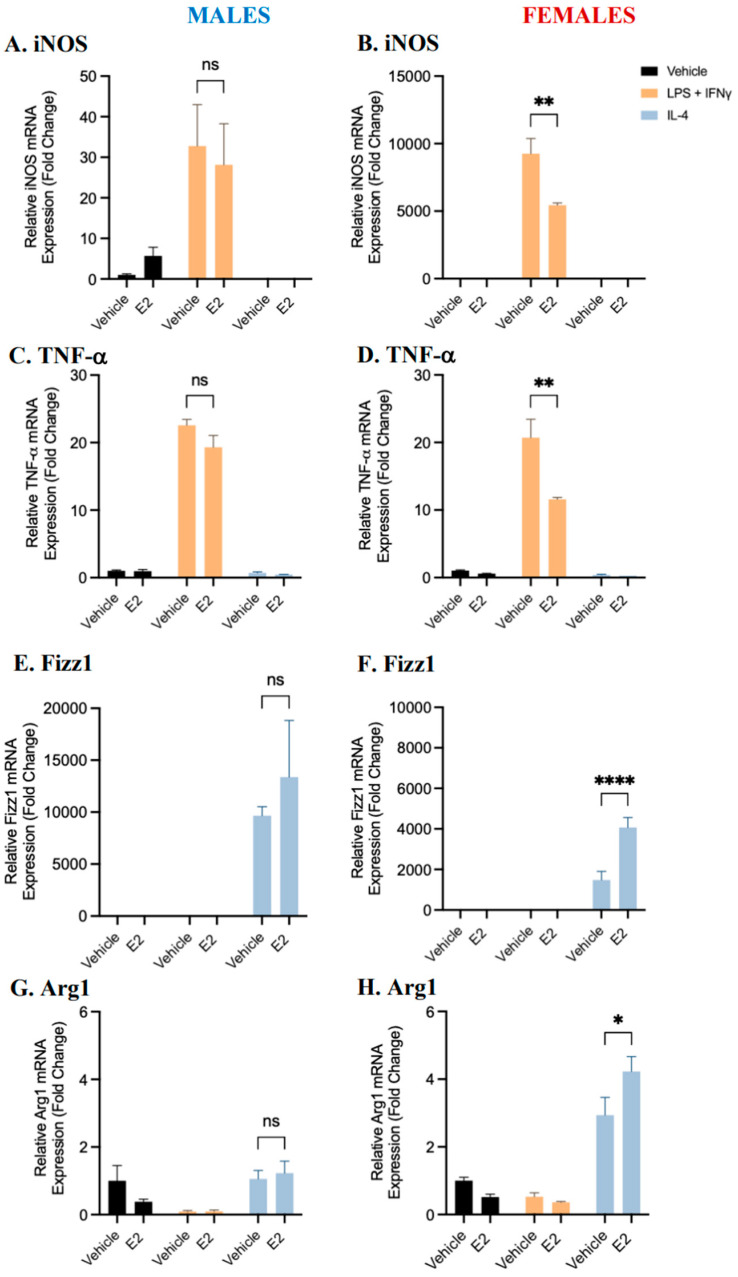
Gene expression of pro- and anti-inflammatory markers in male- (**A**,**C**,**E**,**G**) and female-(**B**,**D**,**F**,**H**) derived BMDMs pre-treated with 100 nM 17β-estradiol (E2). BMDMs were pre-treated with 100 nM E2 for 24 h and polarized to pro- and anti-inflammatory macrophages using LPS and IFNγ, IL-4, or the vehicle control for 24 h. The gene expression of iNOS, TNF-α, Fizz1, and Arg1 from male-derived and female-derived BMDMs 24 h following polarization was quantified by RT-qPCR, as indicated. Data shown represent the mean ± SEM fold change (2^−ΔΔCt^) in marker expression relative to the reference gene β-actin, normalized to the vehicle control. A two-way ANOVA and Tukey’s multiple comparison test were performed to evaluate significant differences (n = 3–5, * *p* < 0.05, ** *p* < 0.01, and **** *p* < 0.0001, ns—not significant).

Treatment with estrogen resulted in a significant reduction in iNOS and TNF-α mRNA expression compared to vehicle controls in pro-inflammatory macrophages derived from female, but not male, BMDMs (Figure 4). A (non-significant) trend towards a reduction in pro-inflammatory marker expression in response to estrogen treatment was observed in male-derived BMDMs. In anti-inflammatory macrophages derived from female mice, treatment with estradiol significantly increased the mRNA expression of Fizz1 and Arg1 compared to the vehicle control. No significant differences were observed in male-derived BMDMs, but a trend towards an increase in anti-inflammatory marker expression was observed following estrogen treatment compared with the vehicle control. These data suggest that estrogen may alter polarization to both pro- and anti-inflammatory phenotypes and may affect male- and female-derived BMDMs differently, or to a different extent.

**Figure 5:**
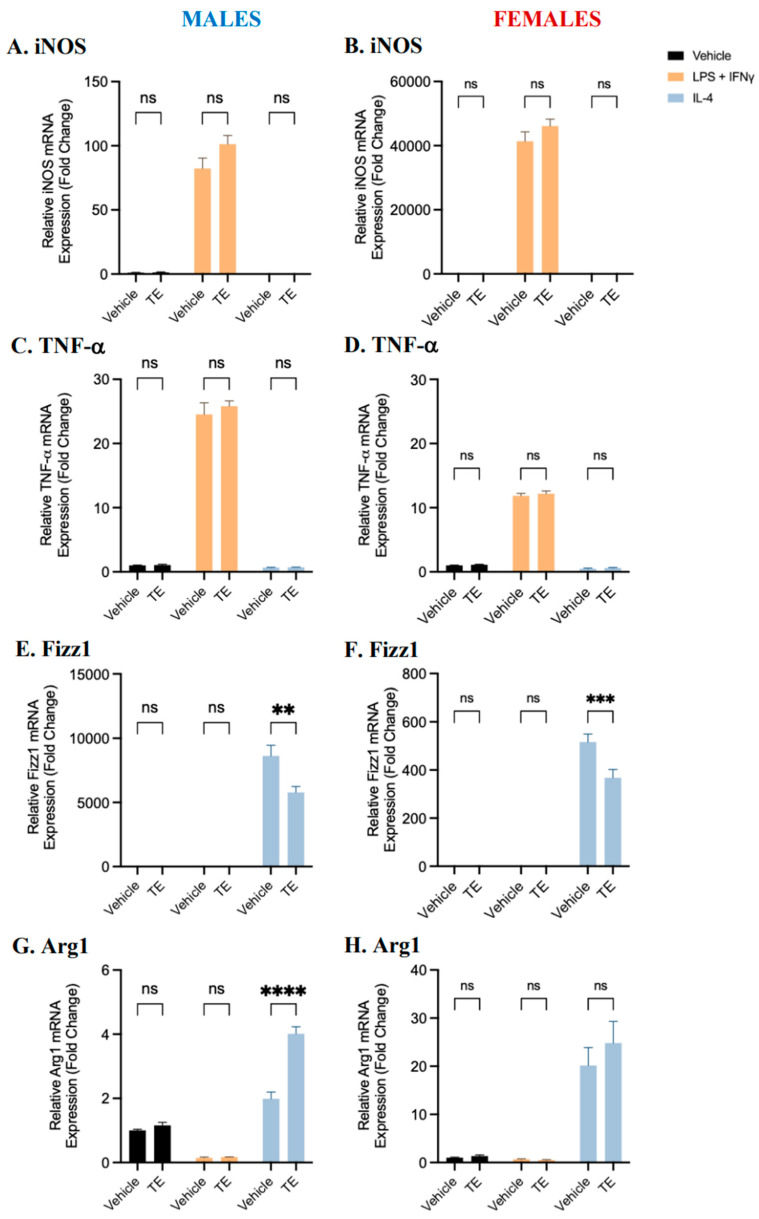
Gene expression of pro- and anti-inflammatory markers in male- (**A**,**C**,**E**,**G**) and female-(**B**,**D**,**F**,**H**) derived BMDMs pre-treated with 100 nM testosterone (TE). BMDMs were pre-treated with 100 nM TE for 24 h and polarized to pro- and anti-inflammatory macrophages using LPS and IFNγ or IL-4, respectively, or the vehicle control, for 24 h. The gene expression of iNOS, TNF-α, Fizz1, and Arg1 from male-derived and female-derived BMDMs 24 h following polarization was quantified by RT-qPCR, as indicated. Data shown represent the mean ± SEM fold change (2^−ΔΔCt^) in marker expression relative to the reference gene β-actin, normalized to the vehicle control. A two-way ANOVA and Tukey’s multiple comparison test were performed to evaluate significant differences (n = 3–5, ** *p* < 0.01, *** *p* < 0.001, and **** *p* < 0.0001, ns—not significant).

Treatment with testosterone did not significantly impact iNOS or TNF-α expression in female-, or male-, derived pro-inflammatory BMDMs compared to vehicle-treated controls (Figure 5). A trend of increasing pro-inflammatory marker expression with testosterone supplementation can be observed in these cells. Testosterone treatment significantly downregulated Fizz1 mRNA expression in anti-inflammatory BMDMs compared to vehicle controls. Conversely, testosterone treatment significantly upregulated Arg1 mRNA expression in anti-inflammatory BMDMs derived from male but not female mice, compared to vehicle controls. These data suggest that testosterone treatment significantly affects polarization to the anti-inflammatory phenotype and may enhance pro-inflammatory macrophage polarization. Overall, the effects of testosterone on polarization appear to be more complex and testosterone may impact cells derived from male and female mice differently.

### 2.3. Sex Differences and the Effects of Sex Hormone Supplementation on Inflammatory Response 

An inflammatory response is a critical function of macrophages in an atherosclerotic plaque. To assess whether sex is an independent factor affecting the inflammatory response of BMDMs, differentiated BMDMs derived from male or female mice were polarized into pro- or anti-inflammatory macrophages for 24 h, and the protein concentration of secreted cytokines and chemokines in cell culture media was analyzed using an addressable laser bead immunoassay (ALBIA). A comprehensive analysis of all analytes measured in M0, pro-, and anti-inflammatory macrophages is presented as Supplementary Figures S2–S4. Generally, BMDMs derived from female mice displayed a significantly enhanced production of specific cytokines, relative to male-derived BMDMs. 

**Figure 6:**
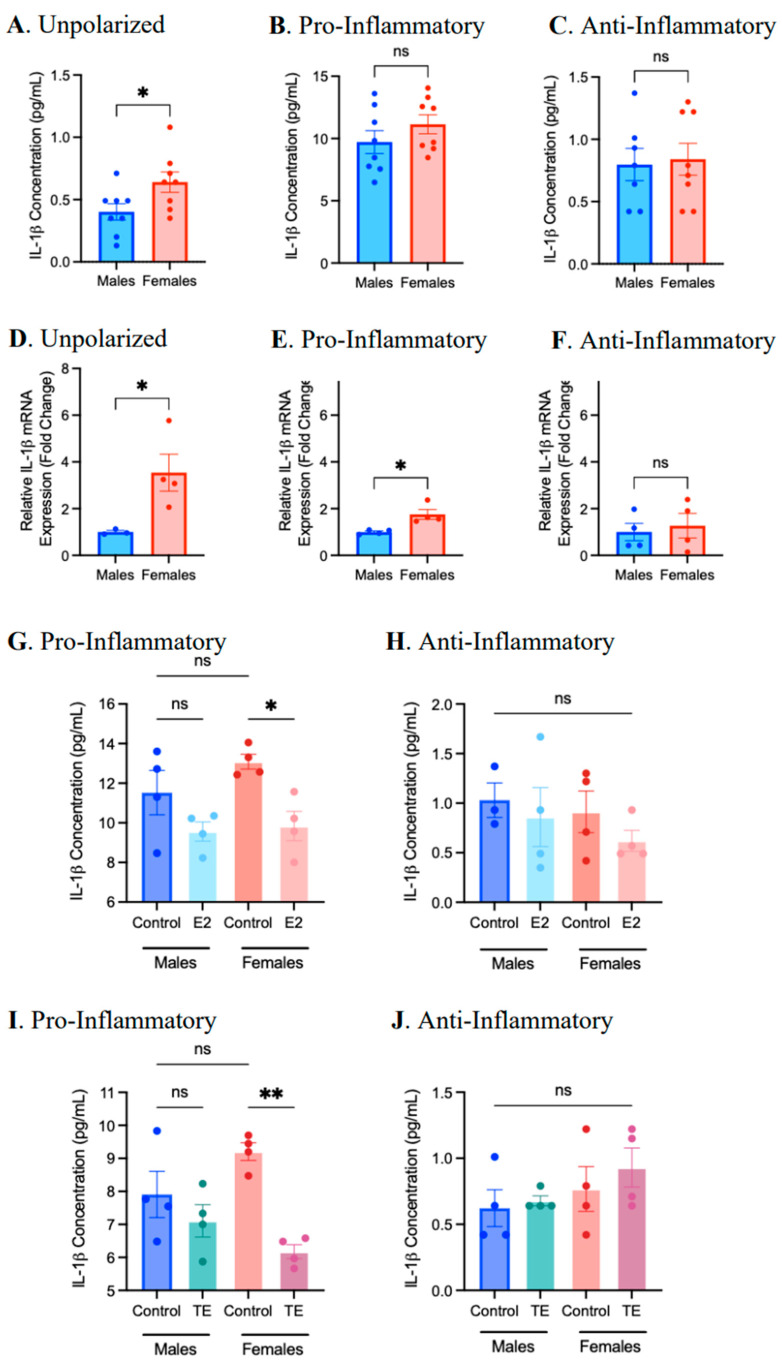
Sex differences and the effects of 17β-estradiol (E2) and testosterone (TE) on IL-1β protein concentration and mRNA expression in BMDMs. (**A**–**F**) Differentiated BMDMs from female and male mice were polarized to pro- or anti-inflammatory phenotypes or left untreated for 24 h. The protein concentration of IL-1β from unpolarized, pro-inflammatory, and anti-inflammatory BMDM culture media was quantified using an addressable laser bead immunoassay (ALBIA). The gene expression of IL-1β from unpolarized, pro-inflammatory, and anti-inflammatory BMDMs was quantified by RT-qPCR. The fata shown are the mean ± SEM fold change (2^−ΔΔCt^) in IL-1β expression relative to the reference gene β-actin, normalized to the vehicle control (**A**–**C**) or mean ± SEM IL-1β protein concentration (**D**–**F**). Significant differences were detected using two-tailed unpaired *t*-tests. (**G**–**J**) Differentiated BMDMs were pre-treated with 100 nM E2, TE, or the vehicle control for 24 h prior to polarization. The data shown are the mean ± SEM IL-1β protein concentration from the cell culture medium. A one-way ANOVA and Tukey’s post hoc test were used to evaluate significant differences. (n = 4–8, * *p* < 0.05, ** *p* < 0.01, ns—not significant).

As IL-1β is a key inflammatory mediator in atherosclerosis [29], we focused our analysis on this cytokine. Differentiated BMDMs were pre-treated with 17β-estradiol (100 nM) or testosterone (100 nM) for 24 h prior to polarization to the pro-inflammatory or anti-inflammatory phenotype. The protein concentration of cytokines and chemokines present in culture media was analyzed using an ALBIA, and the mRNA expression of IL-1β in vehicle controls was compared. As previously observed (Supplementary Figures S2–S4), the IL-1β protein concentration was significantly elevated in M0 macrophages derived from female mice, but no significant sex differences in IL-1β expression were observed in pro- or anti-inflammatory macrophages (Figure 6). Of note, IL-1β mRNA expression in female-derived M0 and pro-inflammatory BMDMs was significantly elevated compared to male-derived BMDMs, thereby corroborating the ALBIA data. Estrogen and testosterone treatment resulted in a significant reduction in IL-1β production in female-, but not male-, derived pro-inflammatory macrophages. Overall, biological sex may represent an independent factor affecting the pro-inflammatory response, and both estrogen and testosterone may alter the production of IL-1β in pro-inflammatory, female-derived BMDMs.

**Figure 7:**
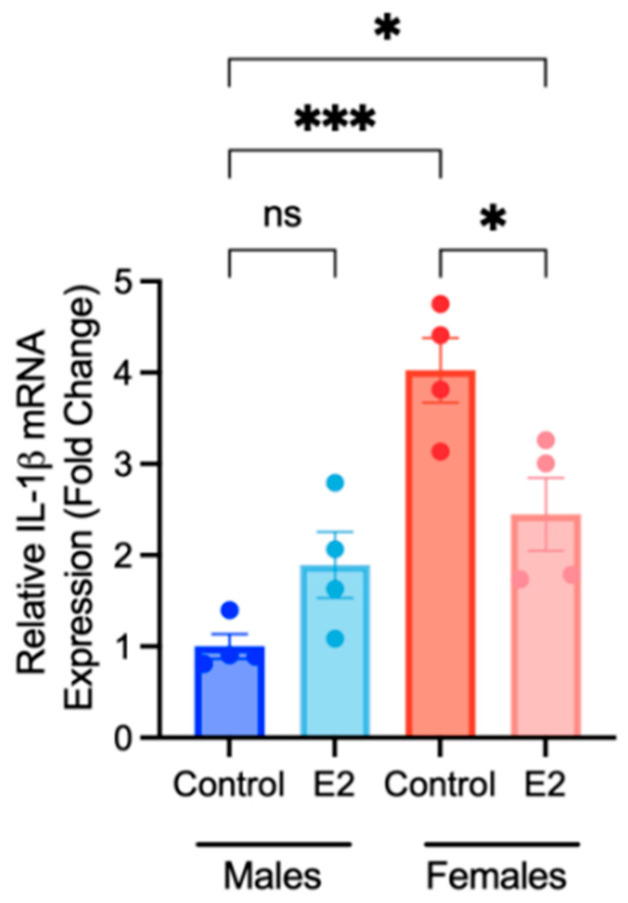
Effects of 17β-estradiol (E2) exposure on IL-1β expression in BMDMs. Isolated bone marrow monocytes were differentiated into BMDMs with or without daily treatments of E2 (100 nM). The gene expression of IL-1β was quantified by RT-qPCR. The data shown are the mean ± SEM fold change (2^−ΔΔCt^) in IL-1β expression relative to the reference gene β-actin, normalized to the vehicle-treated male control. A one-way ANOVA and Tukey’s post hoc test were used to evaluate significant differences. (n = 4, * *p* < 0.05, *** *p* < 0.001, ns—not significant).

Due to the use of estrogen-free cell culture conditions in the differentiation of BMDMs, it was unclear if the observed increases in the expression and abundance of pro-inflammatory cytokines by female-derived BMDMs was a result of true sex differences in this model, or rather due to the deprivation of endogenous estrogen that female-derived cells experienced in culture during the course of our experimental protocol. To address this question, bone marrow was collected from male and female mice, and the same bone marrow monocyte differentiation protocol was carried out with the addition of daily treatments of 100 nM 17β-estradiol or a control. The addition of daily estradiol treatment represented exposure to endogenous estrogen. Differentiated macrophages were collected, and the gene expression of IL-1β was measured using RT-qPCR. Estradiol supplementation for 6 days resulted in a significant reduction in IL-1β expression in female-derived BMDMs (Figure 7). Control (untreated) female BMDMs demonstrated significantly greater expression of IL-1β compared to male control-treated BMDMs (*p* < 0.0001); however, while IL-1β expression in female estradiol-treated BMDMs was significantly greater than in male control-treated BMDMs, the significance level of this comparison was lower (*p*-value = 0.0395). These data suggest that experimental conditions did effect IL-1β expression; however, there is a true effect of biological sex on this system.

### 2.4. Sex Differences and the Effects of Sex Hormone Supplementation on the Migratory Response

**Figure 8:**
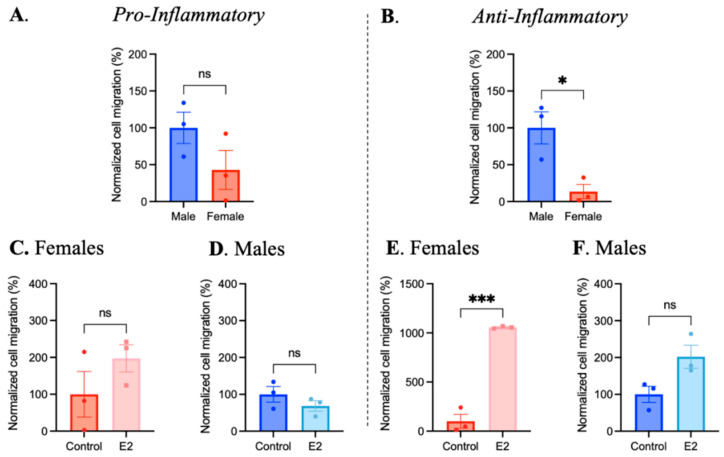
Sex differences and the effects of 100 nM 17β-estradiol (E2) on BMDM migration. Differentiated BMDMs from female and male mice were polarized to pro- or anti-inflammatory phenotypes. A transwell assay was performed with or without chemoattractant CCL19 in the culture medium of the lower assay chamber. (**A**,**B**) The data represent the mean ± SEM cell migration (%) of female pro-inflammatory or anti-inflammatory BMDMs, normalized to male BMDMs. (**C**–**F**) Differentiated BMDMs were pre-treated with 100 nM E2 for 24 h prior to polarization. Following the same transwell protocol, the mean ± SEM cell migration (%) of pro-inflammatory or anti-inflammatory BMDMs, normalized vehicle-treated controls, was calculated. Two-tailed, unpaired *t*-tests were performed to evaluate significant differences (n = 3–4, * *p* < 0.05, and *** *p* < 0.001, ns—not significant).

Migration is an important function of macrophages in an atherosclerotic plaque [15]. To assess whether sex is a factor affecting macrophage migration, BMDMs derived from male and female mice were polarized and a transwell migration assay was performed. Normalized migratory values were created by accounting for passive migration. No sex differences were observed in the normalized number of pro-inflammatory macrophages migrated (Figure 8); however, significantly fewer anti-inflammatory macrophages derived from female mice migrated compared to male-derived BMDMs.

To address this observed difference, BMDMs were treated with 17β-estradiol (100 nM) or the vehicle for 24 h prior to macrophage polarization and the transwell migration assay. Estradiol treatment did not significantly affect the number pro-inflammatory macrophages that migrated regardless of sex. Interestingly, the number of female-derived anti-inflammatory macrophages migrated was significantly increased by estradiol treatment (Figure 8). Treatment with testosterone (100 nM) did not significantly alter BMDM migration (Supplementary Figure S5). These data indicate that estradiol but not testosterone may promote the migration of anti-inflammatory macrophages.

## 3. Discussion

Biological sex and concentrations of circulating sex hormones play a role in the progression of CVD [1,6,7]. Macrophages are a primary cell type abundant in atherosclerotic lesions, and their phenotypic distribution in a lesion has been correlated with plaque severity [30]. In this study, we hypothesize that sex hormones modulate macrophage polarization and pro-atherogenic function and thereby regulate the progression of atherosclerosis. To address our hypothesis, murine-derived bone marrow was isolated from male and female mice, and bone marrow-derived monocytes were differentiated into BMDMs. BMDMs were polarized into pro- or anti-inflammatory phenotypes. To investigate the effects of sex hormones, BMDMs were pre-treated with 17β-estradiol or testosterone (100 nM), and the gene expression of pro- and anti-inflammatory macrophage markers, the protein concentration of inflammatory mediators, and migratory properties were examined.

Together, our findings suggest that sex differences do exist in macrophage polarization, pro-inflammatory responses, and migration. The results support an athero-protective role for estradiol by reducing polarization to the pro-inflammatory phenotype and promoting polarization to the anti-inflammatory phenotype, as well as by diminishing the inflammatory responses of pro-inflammatory macrophages. Estradiol may also promote the migration of anti-inflammatory macrophages. Furthermore, our data support an athero-protective role for testosterone by promoting anti-inflammatory macrophage polarization and by decreasing the inflammatory responses of pro-inflammatory macrophages; however, the results suggest that the effects of testosterone are more complex compared to estradiol.

Our lab previously demonstrated that 95% of cells subjected to differentiation with M-CSF were F4/80+ and CD11b+ via flow cytometry, which validates the efficiency of our macrophage differentiation protocol [22]. In the present study, we expanded upon this protocol by analyzing the gene expression of multiple pro-inflammatory (iNOS, TNF-α, Il-1β, and CD38) and anti-inflammatory (Arg1, Fizz1, Ym1, and CD206) macrophage markers following polarization.

The direct comparison of macrophage polarization markers in male- and female-derived BMDMs shows that the pro-inflammatory markers TNF-α and Il-1β and the anti-inflammatory markers Fizz1 and Ym1 were significantly elevated in female-derived cells, compared to male-derived cells at the same time point. These data suggest that female-derived BMDMs may be more sensitive to environmental stimuli. These observations are also, at least partially, attributable to the hormone deprivation that occurred as a result of our study protocol (see Figure 7).

The influence of epigenetics and the effect of X and Y chromosomes on pathology are emerging areas of study that might explain the sex differences observed in this study and elsewhere. Recently, it has been suggested that non-resolving inflammation in atherosclerotic lesions could be caused, at least in part, by an impaired phenotypic switch of atherosclerosis-associated macrophages into anti-inflammatory macrophages [31]. These findings relate to the hypothesized epigenetic mechanism, termed macrophage repolarization, in which M1-like macrophages are skewed towards an M2-like phenotype by turning off M1 machinery [32]. Extensively reviewed evidence of estrogen and estrogen-receptor-induced epigenetic mechanisms, including DNA methylation, histone modification, and chromatin remodelling [33], suggest that macrophage polarization in atherosclerosis could be regulated by estrogen and estrogen receptors through epigenetic mechanisms. The number of X chromosomes has also been shown to be a factor in pro-inflammatory cytokine production following Toll-like receptor stimulation in human purified monocytes [34].

The observed sex differences in our study do not appear to arise from a difference in the expression of sex hormone receptors on macrophages. We evaluated the gene expression of androgen receptors, ERα, ERβ, or GPER1, in male- and female-derived BMDMs. Female-derived BMDMs demonstrated a significantly greater expression of androgen receptors, while no significant differences were observed in the gene expression of Erα, ERβ, or GPER1.

Significant differences in the gene expression of iNOS and TNF-α in pro-inflammatory macrophages, and Fizz1 and Arg1 in anti-inflammatory macrophages, in response to estradiol supplementation were only observed in cells derived from female mice. This may further support our interpretation that female-derived cells may be more sensitive to environmental stimuli, especially given that the gene expression of estrogen receptors was not significantly different when comparing male- and female-derived BMDMs. This may also indicate a sex difference in the effects of estrogen on BMDM polarization. Overall, we demonstrated that 17β-estradiol may impair pro-inflammatory macrophage polarization and enhance anti-inflammatory macrophage polarization.

Pro-inflammatory marker expression was not affected by pre-treatment with testosterone, indicating that testosterone does not alter polarization to the pro-inflammatory phenotype. Overall, the effects of testosterone on macrophage polarization to the pro-inflammatory phenotype are unclear, but testosterone may promote polarization to the anti-inflammatory phenotype.

Interleukin-1β (IL-1β) is a pro-inflammatory cytokine that has been identified as both a marker and mediator of inflammation in atherosclerosis [29,35]. For this reason, IL-1β was a primary marker of inflammation in this study. Isolated bone marrow-derived monocytes were supplemented with 100 nM 17β-estradiol daily for 6 days while differentiation into BMDMs using M-CSF was carried out. We observed that daily estradiol treatment dampened IL-1β expression in female-derived M0 macrophages; however, IL-1β expression in these estradiol-treated BMDMs remained significantly greater than in male-derived BMDMs. These data demonstrate that experimental conditions only partially precipitated inflammatory responses in female-derived BMDMs, and that sex differences in the BMDM inflammatory response may still exist.

To further investigate the effects of estradiol or testosterone on BMDM pro-inflammatory responses, we pre-treated BMDMs with estradiol or testosterone prior to polarization and assessed the concentration of IL-1β in cell culture media. We observed that estradiol and testosterone treatment diminished IL-1β production by pro-inflammatory macrophages from female-derived mice with a closely aligned trend observed in BMDMs from male-derived mice. Interestingly, these findings are in line with the anti-atherogenic effect of estradiol on macrophage polarization previously observed in this study and elsewhere [8,17]. In addition, the anti-inflammatory role of testosterone previously suggested [36] is further supported by these findings. Cleaved IL-1β protein levels are largely regulated by the NLRP3 inflammasome through caspase-1 activation [37,38]. The NLRP3 inflammasome also regulates IL-18 protein levels in a similar manner [37,38]. Therefore, determining whether estradiol and testosterone affect NLRP3 mRNA expression and IL-18 production could provide further insights into the mechanism by which estradiol and testosterone regulate IL-1β production.

Migration is a key function of macrophages within atherosclerotic lesions. The migration of pro-inflammatory macrophages allows for localization to areas of cholesterol content, which leads to foam cell formation [39]; however, migratory properties seen in anti-inflammatory macrophages may relate to increased cholesterol efflux and plaque stabilization [39]. We investigated whether any differences exist between male- and female-derived BMDMs in their ability to migrate towards the chemoattractant CCL19. A transwell migration assay was performed with CCL19 supplemented in the lower chamber, and data suggest a significantly lower migratory ability of female-derived anti-inflammatory BMDMs compared to male-derived BMDMs. These data contribute to the trend observed in this study that female-derived BMDMs may be more sensitive to environmental stimuli than male-derived BMDMs. Furthermore, we supplemented BMDMs with estradiol or testosterone prior to performing the transwell assay. In anti-inflammatory macrophages from both male- and female-derived mice, estradiol supplementation increased migration towards CCL19. These data suggest a protective role for estradiol in atherosclerosis by enhancing the migration of anti-inflammatory macrophages.

One line of research suggests that the rate of migration may be related to the expression of integrins α_M_β_2_ and α_D_β_2_ [40]. While integrins α_M_β_2_ and α_D_β_2_ affected pro-inflammatory macrophage migration in an opposing manner, both molecules promote anti-inflammatory macrophage motility. Therefore, it is possible that estradiol actions may regulate integrin molecule expression, which could explain the increase in the migration of anti-inflammatory macrophages observed in our study. More research is needed in this area to better interpret these data. Testosterone supplementation did not appear to alter BMDM migration, despite a previous report that 100 nM testosterone supplementation significantly increased T-cell regulator migration [41].

The primary limitation of this study is the use of BMDMs in vitro as a proxy for macrophages in atherogenesis as well as the application of estradiol and testosterone to these cells, which are both oversimplifications of dynamic and complicated pathological and endocrinological systems. Additionally, our study does not evaluate the effects of progesterone, or the combination and fluctuations (estrous cycle) of sex hormones, all of which are potentially relevant and necessary to examine in future studies. Another limitation of our study is that we were unable to investigate the mechanistic effects of estradiol and testosterone on all proteins. The detection of total and phosphorylated STAT6 and STAT1 was unsuccessful due to the extremely low abundance of total and phosphorylated target proteins. Finally, to support the translational relevance of these findings, it will be important to replicate this study in macrophages isolated from human subjects.

Overall, the results of this study begin to explain the effects of sex hormones on macrophage polarization and function in atherogenesis. Because of the important role that macrophages play in atherosclerosis, observations made from this work will provide insights into the interactions of biological sex and cardiovascular disease. Ultimately, this research supports the development of more effective sex-specific strategies to better treat individuals with CVD.

## 4. Materials and Methods

### 4.1. Bone Marrow-Derived Macrophage Isolation and Polarization

C57BL/6 mice were obtained from Jackson Labs. Mice were provided with unlimited food and water and were housed in a 12 h light/dark cycle. All use of mice was pre-approved by the McMaster University Animal Research Ethics Board and followed guidelines regulated by the Canadian Council of Animal Care. The following bone marrow isolation protocol was carried out as previously described [22]. At ages 8–10 weeks, tibias and femurs were harvested. Bone marrow was collected and passed through a 70 μM nylon filter. Isolated bone marrow was resuspended in phenol red-free Dulbecco’s Modified Eagle Medium (DMEM, Thermo Fisher, Mississauga, ON, Canada) containing 20% charcoal-stripped fetal bovine serum (CS-FBS, Sigma Aldrich, Oakville, ON, Canada), 100 IU/mL penicillin, 100 μg/mL streptomycin, and 1× MEM non-essential amino acids (DMEM plating medium). Cells were seeded onto 10 cm non-treated Petri dishes at a seeding density of 5 × 10^6^ cells/plate in 10 mL of media. To initiate differentiation to M0 macrophages, 20 ng/mL of recombinant macrophage colony-stimulating factor (M-CSF) was applied to each dish [22]. Following 3 days of incubation, 5 mL of fresh medium and 20 ng/mL M-CSF were added to each plate. On day 6, cells were washed twice with warm, sterile PBS without calcium or magnesium. Cells were lifted using Accutase^®^ (Cedarlane, Burlington, ON, Canada), centrifuged at 200× *g* for 5 min, and replated in 12-well tissue culture dishes at a density of 4 × 10^5^ cells/well in 1 mL of the DMEM plating medium. After 24 h of rest, subsets of cells were treated with 100 ng/mL LPS and 20 ng/mL IFNγ to polarize to the pro-inflammatory phenotype [22,25], 10 ng/mL IL-4 to polarize to the anti-inflammatory phenotype [22,26], or left unstimulated in fresh DMEM plating medium with 1× PBS. Sample collection occurred after 6, 24, and 48 h of incubation.

### 4.2. Hormone Pre-Treatment

Bone marrow-derived monocytes were differentiated into macrophages using M-CSF as described above. Two hours following cell replating onto 12-well culture dishes, cells were supplemented with 100 nM 17β-estradiol [16], testosterone [27,28], or PBS as the vehicle control, and incubated for 24 h. The hormone concentrations used are based upon previous publications and preliminary titration experiments. Cells were polarized into their respective phenotypes using the polarization protocol as described, in the presence or absence of hormone treatments, depending on the assay performed.

### 4.3. Analysis of Gene Expression

Bone marrow-derived macrophages were differentiated, pre-treated with estrogen or testosterone, and polarized in 12-well tissue culture dishes at 4 × 10^5^ cells/well in 1 mL of the DMEM plating medium. Following polarization, TRIzol^®^ Reagent was applied to adhered macrophages and total RNA was collected using the TRIzol^®^ Reagent (Thermo Fisher) manufacturer’s protocol. Isolated RNA was resuspended in RNase-free water and quantified using a NanoDrop (Thermo Fisher). The purity and quantity of RNA were determined using A260/A280 and A260/A230 ratios produced by the NanoDrop. cDNA was produced from 1 μg of total RNA that was reverse transcribed using a High-Capacity cDNA Reverse Transcription kit (Thermo Fisher) [22]. A real-time quantitative polymerase chain reaction (qPCR) reaction mixture was created using 12.5 μL of SensiFAST SYBR No-ROX Kit (Thermo Fisher), 1.25 μL of forward and reverse primers (500 nM), 8 μL of RNase-free water, and 2 μL of resultant cDNA. qPCR was performed and the relative fold change (2^−ΔΔCt^) for each target gene was calculated by normalizing data to the reference gene, β-actin. Common markers of pro-inflammatory macrophages include iNOS, TNF-α, IL-1β, and CD38 [16,17,23,24], and anti-inflammatory macrophages have been characterized by the expression of Fizz1, Arg1, Ym1, and CD206 [16,17,23,24]. To maintain consistency with the literature, these markers were employed in our study to characterize pro-inflammatory and anti-inflammatory macrophage phenotypes. The primers used for this reaction were specific for murine iNOS, TNF-α, IL-1β, CD38, Fizz1, Arg1, Ym1, and CD206 (Table 1). Sex hormone receptor expression was quantified using primers specific for ERα, ERβ, GPER1, and androgen receptors (Table 1).

### 4.4. Cytokine/Chemokine Detection

To assess the pro-inflammatory responses of BMDMs, the production of pro-inflammatory cytokines and chemokines was analyzed using an addressable laser bead immunoassay (ALBIA), also referred to as a multiplexing laser bead assay as previously described [22]. Briefly, following BMDM polarization and hormone pre-treatment, cell culture media were collected, centrifuged at 200× *g* for 5 min, and the pellet was discarded. The concentration of cytokines and chemokines in supernatant was quantified using an ALBIA (Mouse Cytokine Array/Chemokine Array 31-Plex (MD31), Eve Technologies, Calgary, AB, Canada).

### 4.5. Macrophage Migration Assay

Following differentiation with M-CSF, BMDMs were seeded into 12-well non-treated tissue culture dishes and were treated with 100 nM 17β-estradiol, testosterone, or the PBS vehicle control for 24 h. Cells were then polarized using LPS and IFNγ or IL-4 in conjunction with 100 nM 17β-estradiol, testosterone, or PBS treatment. The following cell migration protocol was carried out as previously described [22]: After 24 h of incubation, cells were re-suspended in the serum-free DMEM plating medium and incubated for 1 h. Transwell inserts (3 μm pore size, 6.5 mm diameter) were coated with rat tail collagen I (4 mg/mL) for 1 h. Cells were detached using Accutase^®^ and seeded into the upper chamber of the transwell insert at a density of 0.4 × 10^5^ cells/200 μL/insert in the serum-free DMEM plating medium and incubated for 30 min at 37 °C. Chemokine ligand 19 (0.5 μg/mL, CCL19), or DMEM alone, were dispensed into the lower chambers of the transwell support system. Following incubation, loaded transwell inserts were placed into the lower chambers and incubated at 37 °C. After 4 h, cells were rinsed with 1× PBS and fixed with 4% paraformaldehyde (PFA) for 15 min. Cells were rinsed with 1× PBS and stained with 4′,6-diamidino-2-phenylindole (DAPI, 1:5000) for 5 min. Following the 1× PBS rinse, membranes were washed twice with 1× PBS. Non-migrated cells located on the upper surface of the membrane were carefully scraped off using a cotton swab. Migrated cells on the lower surface of the membrane were imaged and quantified using a fluorescent microscope (Olympus BX41 microscope connected to a DP71 Olympus camera, with 10× objective, Olympus Corporation, Tokyo, Japan). For quantification, 4 images were taken of each insert, representing one biological replicate, and DAPI-stained nuclei were counted using ImageJ 1.52q software. The total number of cells migrated per replicate was determined by combining the cell count from each image in a biological replicate (i.e., 4 images combined). Passive migration was accounted for by subtracting the number of cells that migrated in CCL19-free control wells from each biological replicate of the corresponding macrophage phenotype. The data were normalized to either male-derived controls or vehicle-treated controls, depending on the analysis. The data presented are the mean cell migration of three to four biological replicates represented as a percentage of the control.

### 4.6. Statistical Analysis

Statistical analyses were performed using GraphPad Prism 7 software. Data were analyzed using a two-tailed unpaired *t*-test or a one- or two-way analysis of variance (ANOVA) test followed by Tukey’s post hoc test to detect significant differences in sample means between groups. Statistical significance was determined by a *p*-value of less than 0.05.

## Figures and Tables

**Table 1:** Primer sequences for RT-qPCR analyses.

Gene	Forward Primer (5′ → 3′)	Reverse Primer (5′ → 3′)
*β-actin*	GGC ACC ACA CCT TCT ACA ATG	GGG GTG TTG AAG GTC TCA AAC
*iNOS*	CAG CTG GGC TGT ACA AAC CTT	CAT TGG AAG TGA AGC GGT TCG
*TNF-α*	ACC ACA GTC CAT GCC ATC AC	CAC CAC CCT GTT GCT GTA GCC
*Fizz1*	TCC AGC TGA TGG TCC CAG TGA ATA	ACA AGC ACA CCC AGT AGC AGT CAT
*Arg1*	ACC TGG CCT TTG TTG ATC TCC CTA	AGA GAT GCT TCC AAC TGC CAG ACT
*IL-1β*	CTG CTT CCA AAC CTT TGA CC	AGC TTC TCC AGA GCC ACA AT
*CD38*	TTG CAA GGG TTC TTG GAA AC	CGC TGC CTC ATC TAC ACT CA
*Ym1*	AGA AGG GAG TTT CAA ACC	GTC TTG CTC ATG TGT AGT GA
*CD206*	CAG GTG TGG GCT CAG GTA GT	TGT GGT GAG CTG AAA GGT GA
*AR*	TTG CAA GAG AGC TGC ATC AGT T	ACT GTG TGT GGA AAT AGA TGG GC
*ERα*	ACC ATT GAC AAG AAC CGG AG	CCT GAA GCA CCC ATT TCA TT
*ERβ*	TGT GTG TGA AGG CCA TGA TT	TCT TCG AAA TCA CCC AGA CC
*GPER1*	TCA TTT CTG CCA TGC ACC CA	GTG GAC AGG GTG TCT GAT GT

## Data Availability

Data are contained within the article and Supplementary Material.

## Supplementary Materials

The supporting information can be downloaded at: https://www.mdpi.com/article/10.3390/ijms25020951/s1.
