# Supplementary material for: Investigating the Effects of Sex Hormones on Macrophage Polarization
Source: Int J Mol Sci. 2024 Jan 12;25(2):951. doi: 10.3390/ijms25020951 (PMC10816176; doi:10.3390/ijms25020951)
Supplement: Supplementary file 1 [file ijms-25-00951-s001.zip › ijms-2800047-supplementary.pdf]

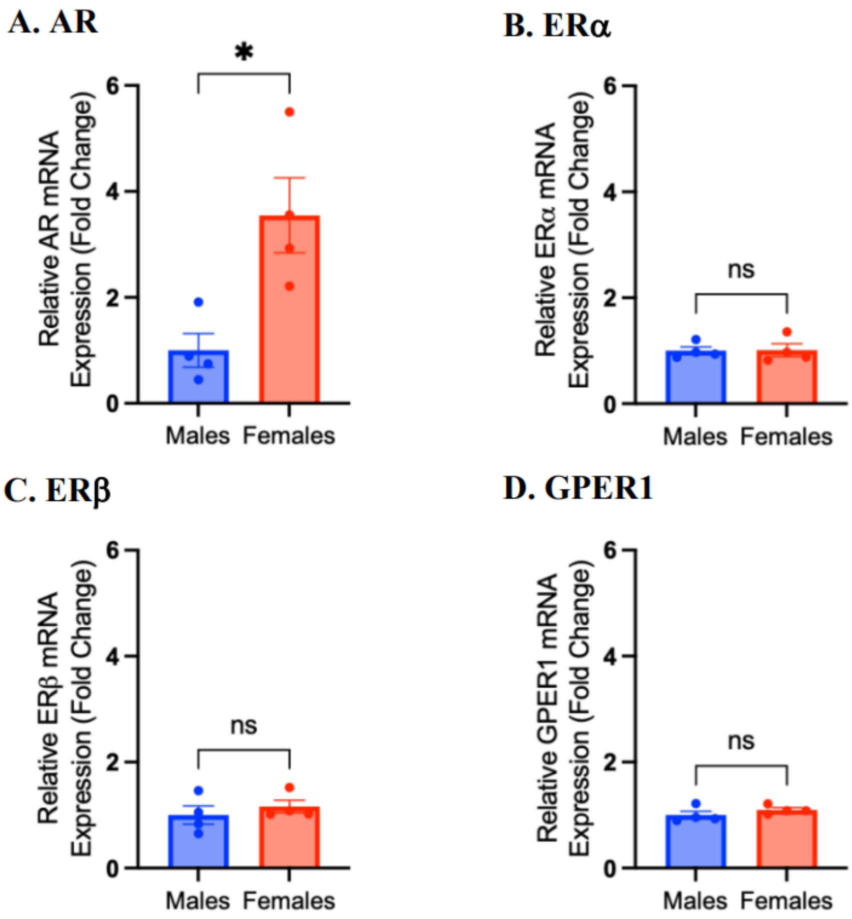

Supplementary Figure S1: Sex differences in gene expression of sex hormone receptors in BMDMs. Bone marrow monocytes from male and female C57BL/6 mice were differentiated into BMDMs. Gene expression of AR, ERα, ERβ, and GPER1 was quantified by RT-qPCR, as indicated. Data shown represent the mean ± SEM fold change (2-ΔΔCt) in receptor expression relative to the reference gene β-actin, normalized to male BMDMs. Two-tailed unpaired t-test was used to evaluate significant differences. (n=4, *p<0.05, ns - not significant).

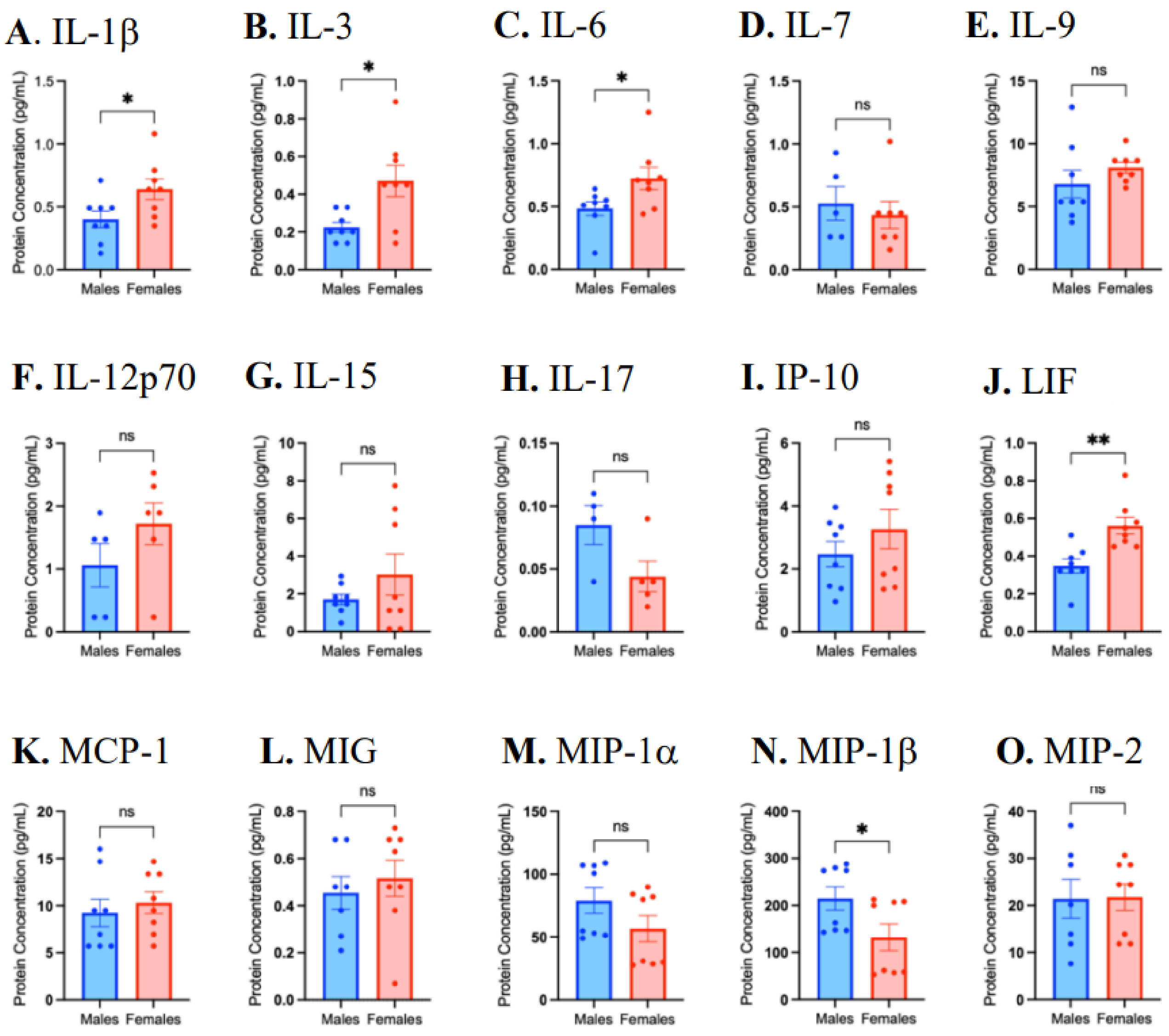

Supplementary Figure S2: Protein concentration of cytokines and chemokines secreted by unpolarized BMDMs derived from male and female mice. Bone marrow monocytes were differentiated into BMDMs, and cells were treated with the vehicle control for 24 hours. Data shown represent the mean ± SEM protein concentration of secreted cytokines and chemokines from cell culture media of BMDMs derived from male and female mice, quantified by an addressable laser bead immunoassay (ALBIA). Two-tailed unpaired t-test was used to evaluate significant differences. (n=5-8, **p*<0.05, ***p*<0.01, ns – not significant).

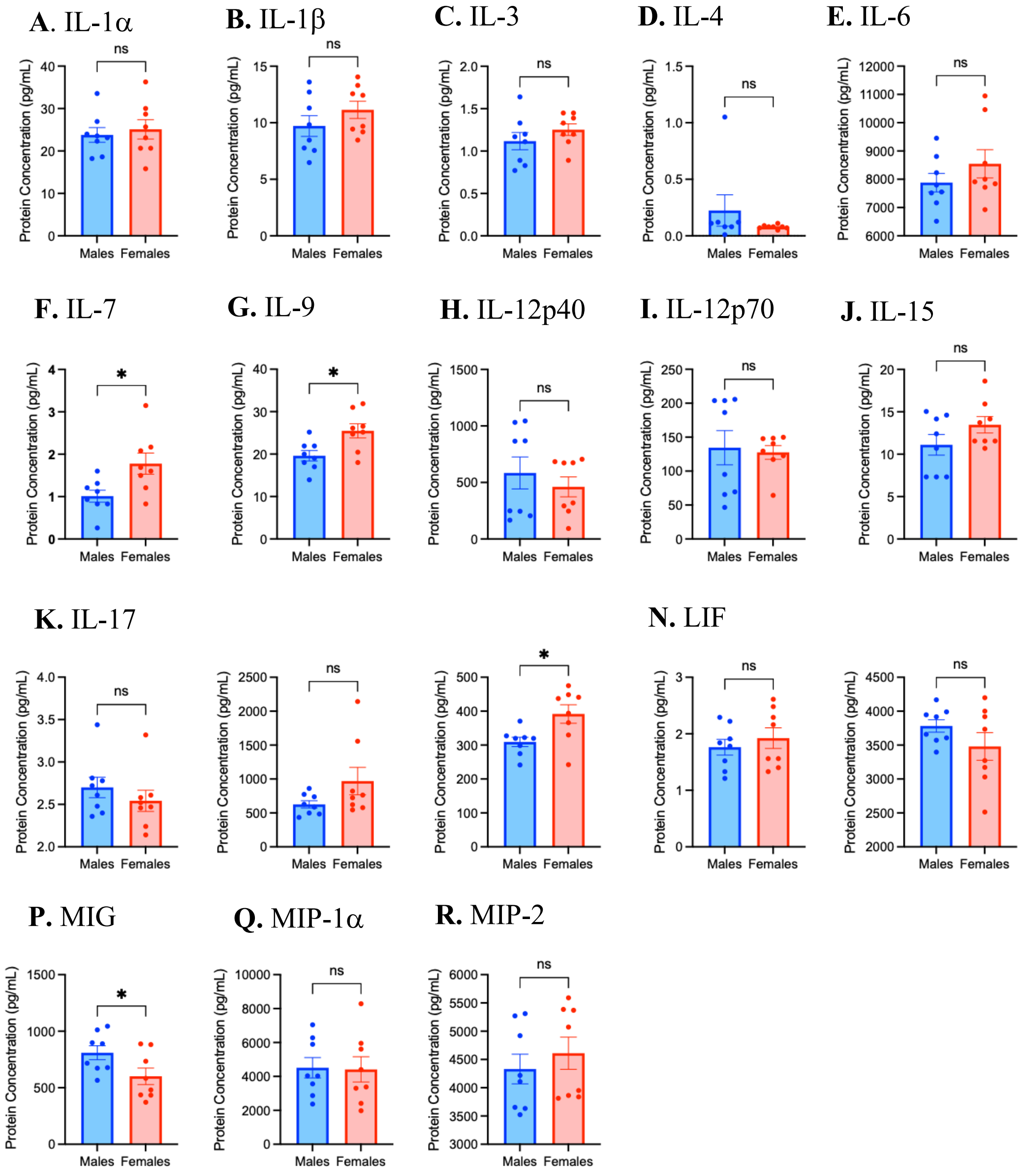

Supplementary Figure S3: Protein concentration of cytokines and chemokines secreted by pro-inflammatory BMDMs derived from male and female mice. Bone marrow monocytes were differentiated into BMDMs, and cells were treated with LPS and IFNγ for 24 hours. Data shown represent the mean ± SEM protein concentration of secreted cytokines and chemokines from cell culture media of BMDMs, quantified by an addressable laser bead immunoassay (ALBIA). Two-tailed unpaired t-test was used to evaluate significant differences. (n=5-8, **p*<0.05, ns – not significant).

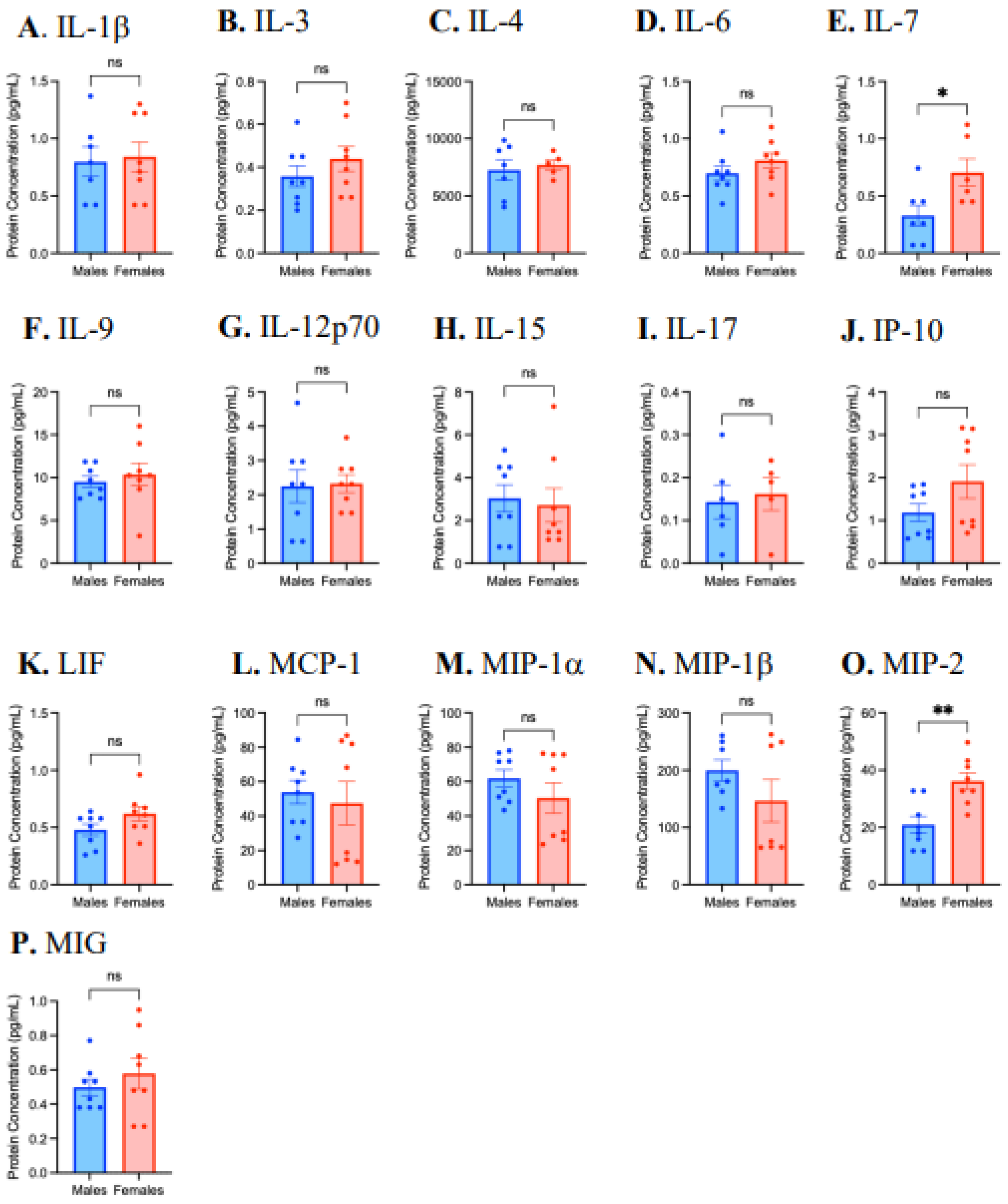

Supplementary Figure S4: Protein concentration of cytokines and chemokines secreted by anti-inflammatory BMDMs derived from male and female mice. Bone marrow monocytes were differentiated into BMDMs, and cells were treated with IL-4 for 24 hours. Data shown represent the mean ± SEM protein concentration of secreted cytokines and chemokines from cell culture media of BMDMs, quantified by an addressable laser bead immunoassay (ALBIA). Two-tailed unpaired t-test was used to evaluate significant differences. (n=5-8, *p<0.05, **p<0.01, ns – not significant).

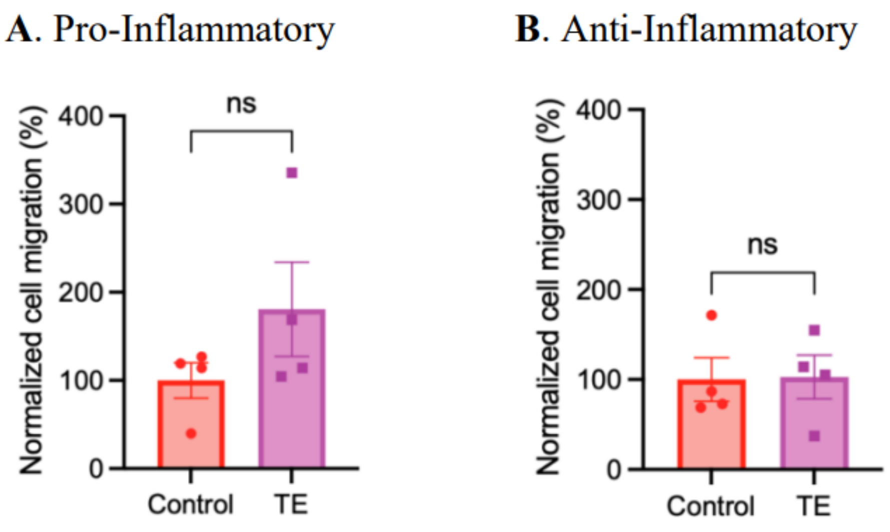

Supplementary Figure S5. The effects of 100 nM testosterone (TE) on BMDM migration. Differentiated BMDMs from female mice were pre-treated with 100 nM TE and polarized to pro- or anti-inflammatory phenotypes. A transwell migration assay was performed with or without chemoattractant CCL19 in culture medium of the lower assay chamber. Data represent the mean ± SEM cell migration (%) of A. pro-inflammatory or B. anti-inflammatory BMDMs, normalized to the vehicle-treated control. Two-tailed, unpaired t-tests were performed to evaluate significant differences (n=4, ns – not significant).
